# Wavelet-Based 3D Reconstruction of Microcalcification Clusters from Two Mammographic Views: New Evidence That Fractal Tumors Are Malignant and Euclidean Tumors Are Benign

**DOI:** 10.1371/journal.pone.0107580

**Published:** 2014-09-15

**Authors:** Kendra A. Batchelder, Aaron B. Tanenbaum, Seth Albert, Lyne Guimond, Pierre Kestener, Alain Arneodo, Andre Khalil

**Affiliations:** 1 CompuMAINE Lab, University of Maine, Orono, Maine, United States of America; 2 Department of Mathematics and Statistics, University of Maine, Orono, Maine, United States of America; 3 Institute for Molecular Biophysics, University of Maine, Orono, Maine, United States of America; 4 Commissariat a l'Energie Atomique, Gif-sur-Yvette, France; 5 Laboratoire de Physique, Ecole Normale Superieure de Lyon, Lyon, France; 6 Centre National de Recherche Scientifique, Ecole Normale Superieure de Lyon, Lyon, France; The University of Chicago, United States of America

## Abstract

The 2D Wavelet-Transform Modulus Maxima (WTMM) method was used to detect microcalcifications (MC) in human breast tissue seen in mammograms and to characterize the fractal geometry of benign and malignant MC clusters. This was done in the context of a preliminary analysis of a small dataset, via a novel way to partition the wavelet-transform space-scale skeleton. For the first time, the estimated 3D fractal structure of a breast lesion was inferred by pairing the information from two separate 2D projected mammographic views of the same breast, i.e. the cranial-caudal (CC) and mediolateral-oblique (MLO) views. As a novelty, we define the “CC-MLO fractal dimension plot”, where a “fractal zone” and “Euclidean zones” (non-fractal) are defined. 118 images (59 cases, 25 malignant and 34 benign) obtained from a digital databank of mammograms with known radiologist diagnostics were analyzed to determine which cases would be plotted in the fractal zone and which cases would fall in the Euclidean zones. 92% of malignant breast lesions studied (23 out of 25 cases) were in the fractal zone while 88% of the benign lesions were in the Euclidean zones (30 out of 34 cases). Furthermore, a Bayesian statistical analysis shows that, with 95% credibility, the probability that fractal breast lesions are malignant is between 74% and 98%. Alternatively, with 95% credibility, the probability that Euclidean breast lesions are benign is between 76% and 96%. These results support the notion that the fractal structure of malignant tumors is more likely to be associated with an invasive behavior into the surrounding tissue compared to the less invasive, Euclidean structure of benign tumors. Finally, based on indirect 3D reconstructions from the 2D views, we conjecture that all breast tumors considered in this study, benign and malignant, fractal or Euclidean, restrict their growth to 2-dimensional manifolds within the breast tissue.

## Introduction

Breast cancer is the most common cancer worldwide according to the World Health Organization (WHO) and the second leading cause of cancer related death among women in the United States. Despite the recent advances in the medical field, the breast cancer rate has continued to increase over the last 30 years. Cancer is easiest to treat when it is found in the early stages of development, making it critical for women to have regular screenings as recommended by the American Cancer Society (ACS). Mammograms are currently one of the most accepted screening processes and are widely recognized to play a vital role in diagnosing the disease. However, the radiological interpretation of mammograms is a difficult task, especially since the mammographic appearance of normal tissue is highly variable. Even though mammograms are typically read by two expert radiologists or a combination of a radiologist with a computer aided detection/diagnosis (CAD) method, a recent study suggests that a minimum of three independent experts combined with a consensus should be used, especially for non-cancer cases [1]. Criticism of the use of screening mammography due to over-diagnosis led some researchers to show that one in three breast cancers identified by mammography would not cause symptoms in a patient's lifetime [2]. Others argue that advanced breast cancer incidence does not support a substantial role for screening in the decrease in mortality [3]. The recent decline in breast cancer mortality would not be due to population-based screening programs, but rather to improved treatments of the sick [4], [5].

This growing wave of criticism of breast cancer screening programs has been synchronous to a similar movement criticizing the increasing use of CAD methods. We hypothesize that this is *not* a coincidence. Indeed, it has been fifteen years since the U.S. Food and Drug Administration approved usage of CAD methods. From 2001 to 2006, CAD prevalence increased from 3.6% to 60.5% and has since increased to about 75% [6]. However, CAD methods are not offering the expected performance [7]. CAD use in screening mammography is associated with decreased specificity [8]. This leads to increased recall rates of healthy women [6], [9], [10] and can cause false-positives on up to 70% of normal cases [11], resulting in an increase of unnecessary stress on women. In a study that compared the effectiveness of a single reader + CAD vs. two independent readers, the results showed that the recall rate for single reader + CAD was higher [12]. And finally, another limitation of existing CAD methods is the inability of the software to rate the significance of the findings [13], similar to the Breast Imaging-Reporting and Data-System (BI-RADS) assessment score [14] given by the radiologist at the time of the mammogram interpretation.

Despite these potentially worrisome signals, the consensus is that the benefits of screening mammography clearly outweigh the disadvantages. In fact, the use of screening mammography is not expected to diminish, but rather to increase. The American Cancer Society (ACS) recommendation is for women age 40+ to have a mammogram every year and to continue to do so for as long as they are in good health. In England, for example, the extension of the breast screening program, from women aged 50–70 to 47–73 is expected to be completed in 2016 [15]. Taken together, this information combined with the results surveyed above indicates that novel computational techniques must be developed, not only to obtain better quantitative mammography reports, but also to better understand the onset and progression of the disease itself.

The fractal dimension is a tool that has been used extensively in all sciences. While standard geometry is limited to the study of so-called Euclidean objects like smooth curves, circles, and cubes, Fractal geometry can be seen as a generalization of Euclidean geometry, where the study of objects exhibiting a geometrical structure that cannot be described with Euclidean tools such as area, perimeter, and volume, becomes naturally characterized, quantitatively, via the fractal dimension. Standard Euclidean geometrical objects have an integer fractal dimension (*D* = 1, 2, 3 for a line, circle and cube respectively), while most (tree-like or filamentary) fractal objects have a non-integer fractal dimension [16].

The link between the fractal nature of malignant growth and pathology in general has been well established [17]–[20]. For example, specific applications of fractal concepts were used to quantitatively characterize skin lesions [21]–[23], the tumor vasculature and the tissue architecture at the cellular level [24]–[26], as well as the epithelia/stroma interface [27], [28]. The application of fractal concepts specifically to the analysis of breast cancer from the study of digitized mammograms is widespread. For example, fractal-based models of (normal) breast tissue background are used to find calcifications by taking the difference between the original mammographic image and the modeled image [29]–[31]. To assess the actual fractal structure of the periphery of the tumor itself, some use the radiologist-identified boundary of masses using different techniques to show that this manually drawn contour is more likely to be smooth (Euclidean) for benign tumors and more spiculated (fractal) for malignant tumors [32]–[34]. In an early study of 25 mammographic cases (10 benign and 15 malignant) investigating the potential utility of the fractal dimension of clusters of microcalcifications as a discriminatory quantifier, 14 out of 15 malignant cases were able to be separated using the fractal dimension [35]. Another study using fractal models with neural networks to detect microcalcifications attempted to quantitatively characterize the clustering of the segmented calcification spots via the fractal dimension. However, the fractal dimension was insufficient to discriminate between actual microcalcification clusters from sets of noise spots, when considering one mammographic view at a time [36].

Since clusters of microcalcifications are one of the most important and often the only indicator for malignant tumors, the reconstruction of the three-dimensional (3D) structure of these clusters from the two mammographic views, cranial-caudal (CC) and mediolateral-oblique (MLO), is critically important. An early attempt at developing a 3D visualization software putting additional diagnostic information at the radiologists disposal was presented fifteen years ago [37]. More recently a joint analysis of two views of the same breast was presented, where the novelty of the scheme includes a two-step matching process: spatial and feature matching. The authors show that the proposed method has advantages of a lower false positive rate compared to the one on a single view [38]. Taken together, this shows that very little has been done in order to quantitatively combine the information obtained from both views, even though it appears to be logical and beneficial from the research. Overall, however, a reliable quantitative assessment methodology in the form of a computer-aided diagnostic system integrating the fractal structure from both mammographic views is lacking. This is what we are proposing in this manuscript, with very promising results.

We propose that the 2D Wavelet Transform Modulus Maxima (WTMM) method [39] has the ability to become a powerful tool in interpreting mammograms. The 2D WTMM method has proven to be successful in almost all fields of applied science, including geology [40], astrophysics [41]–[43], surface science [44], cellular biology [45]–[51] and orthopedic medicine [52]. The method was originally developed as a multifractal formalism to analyze highly complex 1D signals [53], [54], 2D surfaces [40], [55], [56] and 3D surfaces [57]. In particular, the 1D WTMM method was successfully applied to demonstrate the multifractality in physiologic dynamics and its breakdown with disease [58]–[62]. A preliminary approach demonstrating the potential of the 2D WTMM method to detect microcalcifications and to quantitatively assess and discriminate between fatty vs. dense tissue was first presented in 2001 [63].

The results presented in this paper were obtained by using the WTMM method on screening mammograms that were taken from an online databank. They demonstrate how this tool could be used as a possible Computer-Aided *Detection* method as well as a Computer-Aided *Diagnostic* method. After all images were analyzed using this method, the fractal information independently obtained from each of the two mammographic views of the same breast was combined to indirectly infer a 3D estimate of the geometrical structure of the breast tumor. A statistical analysis was performed on all data, which provided us with information on the critical differences between the organization of both benign and malignant tumors. Importantly, both views of the same breast (CC and MLO) were analyzed independently in terms of microcalcification detection and fractal dimension estimation. The differences in fractal dimension between benign vs. malignant MC clusters were very significant when using population-based statistics. However, in order to develop a diagnostic scheme that would provide high accuracy categorization on a case-by-case basis, the fractal dimension of the cluster of microcalcifications from both views was integrated into a two-coordinate score that was used to plot each case in the “CC-MLO fractal dimension plot”. In this plot we have defined a “fractal zone” consisting almost exclusively of malignant cases and “Euclidean zones” (non-fractal) consisting almost exclusively of benign cases. Therefore, as a potential diagnostic tool, this methodology could be used to assist radiologists by calculating an assessment based on a particular case's position in the CC-MLO fractal dimension plot, and whether or not it is inside or outside the fractal zone. It can do this with high accuracy and in a manner that is independent of the density of the background tissue. Furthermore, the fractal vs. Euclidean distinction between malignant vs. benign tumors, could lead to a better understanding of the biophysical processes that drive tumor growth.

## Results

### Detection of MC Clusters and Calculation of their Fractal Dimension

**Figure 1:**
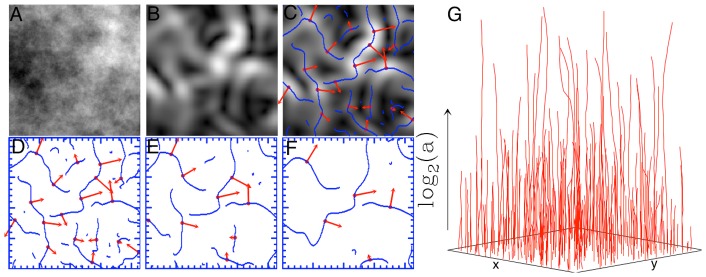
The 2D WTMM Method. (A) Sample simulated fractional Brownian motion image **B**_H = 0.5_(**x**) [39]. (B) The gradient of the image in (A) is obtained as the modulus of the wavelet-transformed using Eq. (6). (C) Maxima chains in blue are defined as positions where the WT modulus is locally maximal (i.e., the WTMM). Along these WTMM chains in (C), further local maxima are found in red, i.e. the WTMMM. This is repeated as several different scales, three of which are shown in (D), (E), and (F). The WTMMM are then connected vertically through scales to define the WT skeleton shown in (G). Gray-scale coding is from black (min) to white (max).

**Figure 2:**
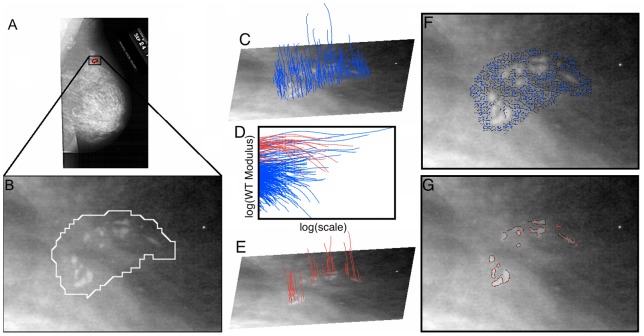
Sample 2D WTMM analysis of a malignant breast lesion. (A) Original image obtained from the DDSM database, where (B) shows the zoomed in image of the radiologist encircled suspicious region. By selecting appropriate values for the slope *h* of the WT modulus as a function of scale in a logarithmic representation, and the log of the pre-factor, log(*K*) in (D) (also see Eq. (1) in the main text that describes this relationship), the WTMMM (blue) from the tissue background in (C) are distinguished from the WTMMM (red) that belong to the MC in (E). From here the WT skeleton can be calculated from the WTMMM that belong to the lesion from those that belong to the background tissue. The corresponding WTMM chains at the smallest scale are shown in (F) and (G) for the background and lesion, respectively.

**Figure 3:**
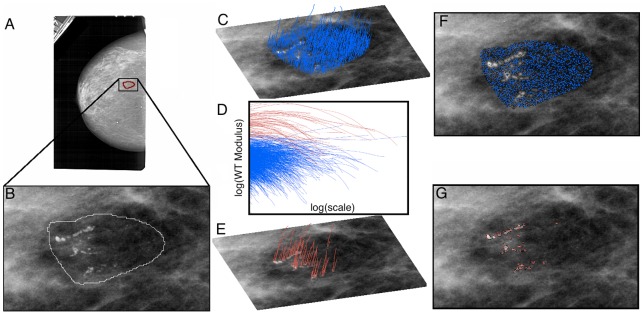
Sample 2D WTMM analysis of a second malignant breast lesion. Same analysis as presented in Figure 2, but on a different case.

As described in detail in the Methods section and in Figure 1, the wavelet transform (WT) acts as a mathematical microscope to characterize spatial image information over a continuous range of size scales. It is the gradient vector of the image smoothed by dilated versions of a Gaussian filter [39], [64]. At each size scale, the wavelet transform modulus maxima (WTMM) are defined by the positions where the modulus of the WT is locally maximal. These WTMM are automatically organized as maxima chains at the considered scale. Along each of these chains, further local maxima are found, i.e., the WTMM maxima (WTMMM). This process is repeated for all size scales and the WTMMM from each scale are then linked to form the WT skeleton. As shown in Figures 2 and 3, the ability to consider (vertical) space-scale WTMMM lines in the WT skeleton individually is key, since it allows us to objectively discriminate between lines pointing to the tissue background from those pointing to the microcalcifications by considering how the WT modulus varies as a function of the scale parameter along each space-scale line. In Figures 2D and 3D, each space-scale line obtained from the WT skeleton is represented by plotting the evolution of the WT modulus, 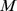 (see Eq. (6) in the Methods section), as a function of the scale parameter, *a,* in a log-log plot. This relationship between 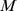 and *a* is characterized by a power-law behavior via the equation:
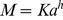
(1)where *K* is a pre-factor and *h* is the Hölder exponent quantifying the strength of the singularity to which the space-scale line is pointing to. In log-log plots shown in Figures 2D and 3D, the slope of the curves therefore corresponds to *h*. By considering two types of information characterizing the behavior of a space-scale line, namely the strength of the modulus at the smallest scale, which is given by the log of the pre-factor, log(*K*), as well as the slope (in a logarithmic representation) of the modulus variation across scales, *h*, a classification procedure is setup which results in two sets of space-scale lines that clearly segregate MC from background tissue. Isolated MC can be seen as Dirac-like singularities through the optics of the WT, for which *h* is theoretically known to be −1 [39]. However, while clustered MC may not appear as isolated Dirac-like singularities, the edge that they form is still easily detectable through the space-scale lines, with a value of *h* ∼ 0 (discontinuity). This means that for both isolated and clustered MC, we can expect the space-scale lines to behave as Eq (1) with *h*≤0, which contrasts from the healthy background tissue, for which *h*∼1/3 for fatty breast tissue and *h*∼2/3 for dense breast tissue [63]. However, relying only on *h* may not be sufficient, which is why the strength of the WT modulus at the lowest scale, which quantifies the contrast between MC and background, is also needed. For the sample case presented in Fig. 2, the plot in Fig. 2D shows that neither log(*K*) nor *h*, taken individually, would have been sufficient to segregate between MC and background. However, for the sample case presented in Fig. 3, the plot in Fig. 3D shows that log(*K*) alone was sufficient. A more detailed discussion of both cases follows.

In Fig. 2 the background breast tissue is dense, which makes the contrast between background and MC weak (i.e. causing a low value for the WT modulus of red curves at the smallest scale in Fig. 2D). However, the roughness fluctuations of dense breast tissue are characterized by a relatively high smoothness level, which translates to blue curves with a large slope (i.e., a high *h* value, ∼2/3) for scales 10≤*a*≤200 pixels as compared to the red curves with negative slopes for scales *a* ≥ 10 pixels that correspond to WTMMM lines that point to MC at small scale (*a*→0^+^) (Fig. 2D) [63]. In Fig. 3, the background breast tissue is fatty, which is characterized by a higher roughness level (i.e. a lower *h* value ∼1/3, although still positive) [63], that reduces the discriminatory power of *h*. However, for MC embedded into fatty tissue, the contrast is high, which translates to a high value of log(*K*). Therefore, applying a threshold on both parameters, *h* and log(*K*), is key to segregating MC from their background regardless of the density (fatty or dense) of the composition of the breast tissue. Given the exploratory nature of this study, the thresholds on *h* and log(*K*) were allowed to vary from one image to another and they were set manually. However, the variability of these thresholds is rather small (data not shown), which shows great potential for a future automation of this processing step.

Once this segregation is done, the so-called singularity spectrum (see Methods section) can then be calculated separately for each subset, which then allows us to consider the fractal dimension *D* (Eq. (16)) of the lesion, characterizing its architecture.

**Figure 4:**
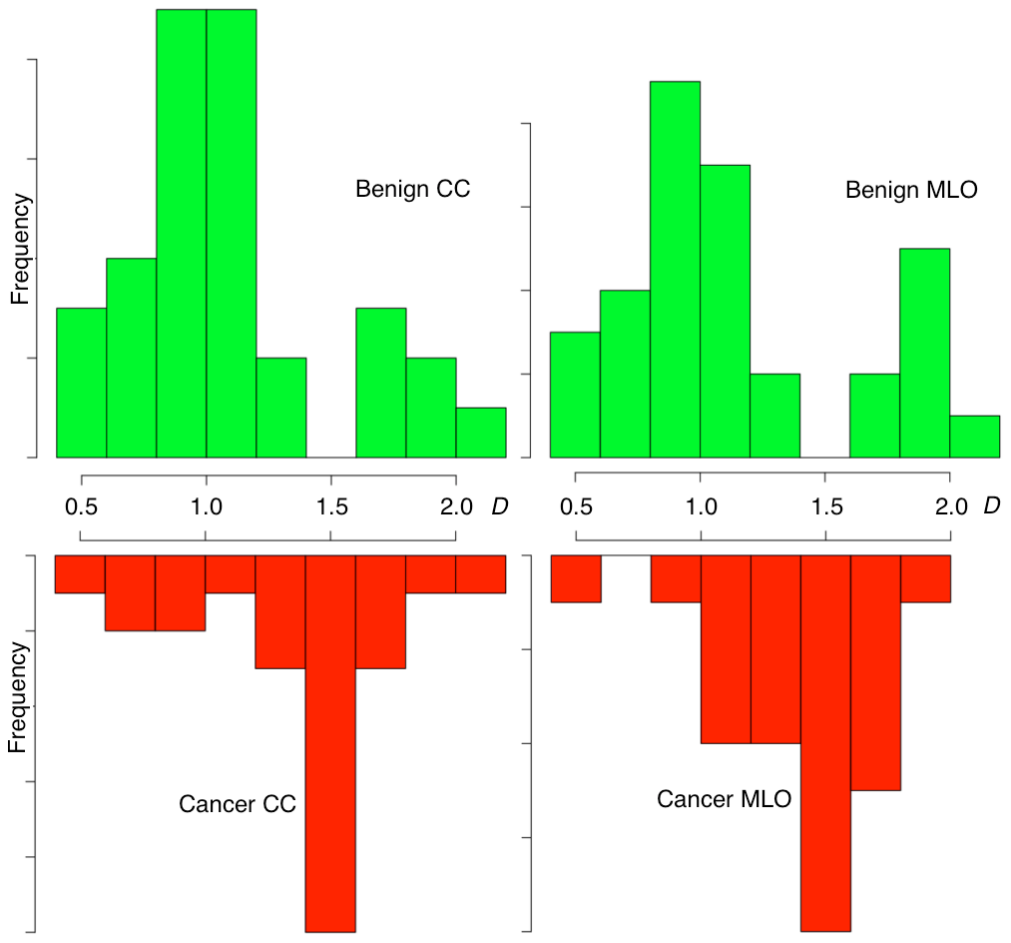
Histograms of fractal dimension values. The frequency distributions of fractal dimensions, *D* (Eq. (16)) calculated for benign CC and MLO views (top) are drastically different than those calculated for the cancer CC and MLO views (bottom).

We restricted the analysis of DDSM cases (see Methods section) to images having a radiologist encircled region that was larger than 256^2^ pixels for both views (CC and MLO) and also to make sure that the distribution of patient ages was comparable (i.e. 56.7+/−11.4 years old for the benign cases and 65.5+/−12.4 years old for the malignant cases). This resulted in an analyzed sample with a total of 59 cases (118 images), 34 of which are benign (68 images) and 25 of which are malignant (50 images). The histograms of fractal dimension values obtained are presented in Figure 4. Note that blending the CC and MLO fractal dimensions together in these distributions would not guarantee an unbiased statistical analysis, which is why the fractal dimension values for the CC and MLO results were analyzed independently. Figure 4 demonstrates that benign MC clusters have a strong preference for Euclidean dimensions that are either close to *D* = 1 or to *D* = 2 and that there is a very clear zone of avoidance in the fractal range, i.e., for 1<*D*<2, with an actual gap in the benign histograms for the bin centered at *D* = 1.5 for both views. For the malignant cases, it is the opposite: Euclidean zones are avoided and the data are very clearly centered in the fractal range for both views, with the peak of the histograms at *D* = 1.5. A statistical comparison between benign vs. malignant MC clusters was performed using the Wilcoxon rank-sum test, which yielded *p*-values of 0.009 for CC comparisons and 0.014 for MLO comparisons for the benign vs. malignant fractal dimension distributions. These are statistically significant differences.

### The CC-MLO Fractal Dimension Plot and the Fractal Zone

**Figure 5:**
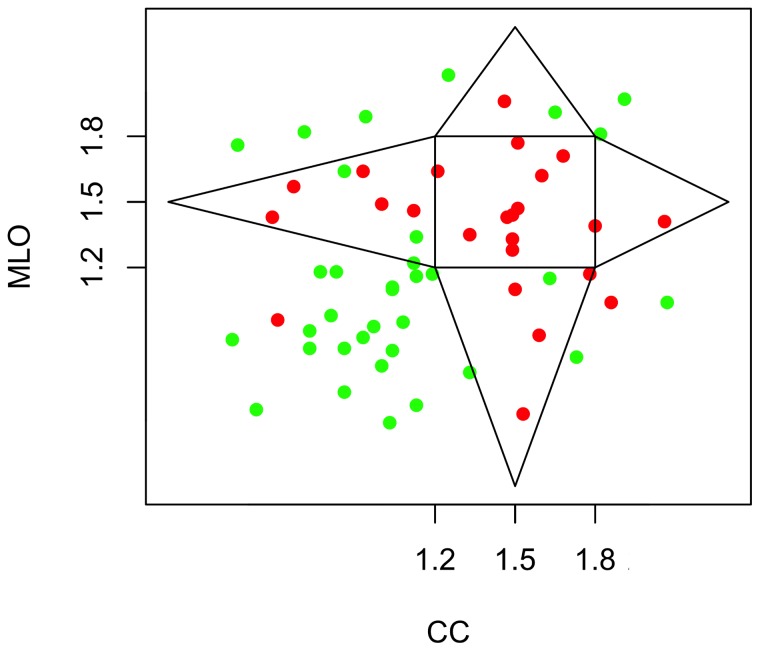
The CC-MLO fractal dimension plot. Each case analyzed is plotted with the fractal dimension obtained from the MLO view as a function of the fractal dimension obtained from the CC view. A polygonal region is outlined, the inside of which is defined as the “fractal zone” while the outside is defined as the “Euclidean zone”. The dots represent malignant (red) and benign (green) cases.

**Figure 6:**
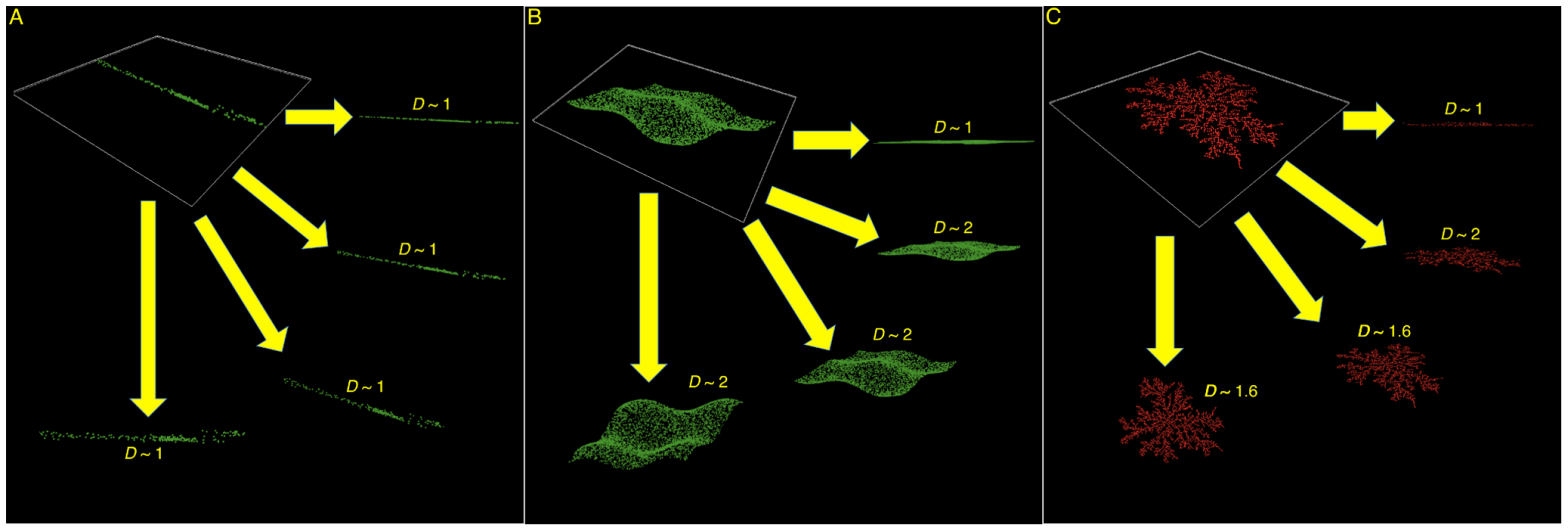
Inferring the 3D geometrical structure of breast lesions based on projection angles. Simulations of random point distribution models are shown to describe how different types of objects, namely a line (A), a surface (B), and a fractal cluster (simulated diffusion-limited aggregate, C) can have only a limited number of possible fractal dimension as a function of the projection angle.

The significance of the difference between benign and malignant is quite interesting. However, it is still only based on statistics of populations. The histograms in Figure 4 show that, when each view is taken independently (CC or MLO), it is still possible, though unlikely, for a malignant lesion to have a Euclidean dimension, and vice-versa, for benign lesions to have a (non-integer) fractal dimension. However, in order to work towards a potential CAD method that would be able to diagnose breast lesions individually instead of via the population statistics, we combined the information to indirectly infer the 3D structure of the tumor embedded into the breast tissue. This is presented in a novel plot called the “CC-MLO fractal dimension plot” shown in Figure 5, where red dots represent malignant cases and green dots represent benign cases. The square centered at (1.5, 1.5) represents those cases for which both CC and MLO views have a fractal dimension that is within 1.2<*D*<1.8. Note that only malignant cases are found in this internal square. However, having one of the two views with a score that is close to *D* = 1.5 should “compensate” for its other view being outside of the [1.2, 1.8] × [1.2, 1.8] square, i.e., as one view approaches *D* = 1.5, the farther from 1.5 the other can be. Further justification is presented below and in Fig. 6. That is how the triangular regions that decay linearly as a function of distance from the internal square were defined. Therefore, the central square, combined with the four triangular regions extending from it are what we define as the “fractal zone”. Of the 59 cases considered in this study, 92% of malignant breast lesions studied (23 out of 25) were in the fractal zone while 88% of the benign lesions were in the Euclidean zones (30 out of 34).

### Bayesian analysis

The inferences from a Bayesian analysis are richer and more informative than null hypothesis significance testing. In particular, there is no reliance on *p*-values. But also, Bayesian models are designed to be appropriate to the data structure without having to make approximation assumptions typical in null hypothesis significance testing [65]. The results reported above obviously show that the vast majority of malignant breast lesions are fractal, and that the vast majority of benign breast lesions are Euclidean. However, the condition of interest is how breast lesions in the fractal zone can indicate malignancy, and how breast lesions in the Euclidean zone can indicate benignancy.

Bayesian inference derives the posterior probability as a consequence of two antecedents, a prior probability and a likelihood function derived from a probability model for the data to be observed. In this application, the model is based on historical radiology assessment scores using the BI-RADS system [14], [66], [67]. A detailed description of this probability model as well as the mathematical model behind Bayes analysis is presented in the Methods section. Bayesian inference then becomes a computation of the posterior probability according to Bayes' rule. The interpretable output of this Bayesian analysis is the so-called 95% highest density interval (HDI), which is analogous to the 95% confidence interval in frequentist statistics. The 95% HDI from the resulting posterior distribution indicates that the percentage of breast lesions in the fractal zone that are malignant is between 74.2% and 97.5%. Alternatively, in terms of controlling for false positives, which is a major concern, as discussed in the Introduction, the percentage of breast lesions in the Euclidean zone that are benign is between 75.7% and 96.2%.

### Additional statistical analysis

**Figure 7:**
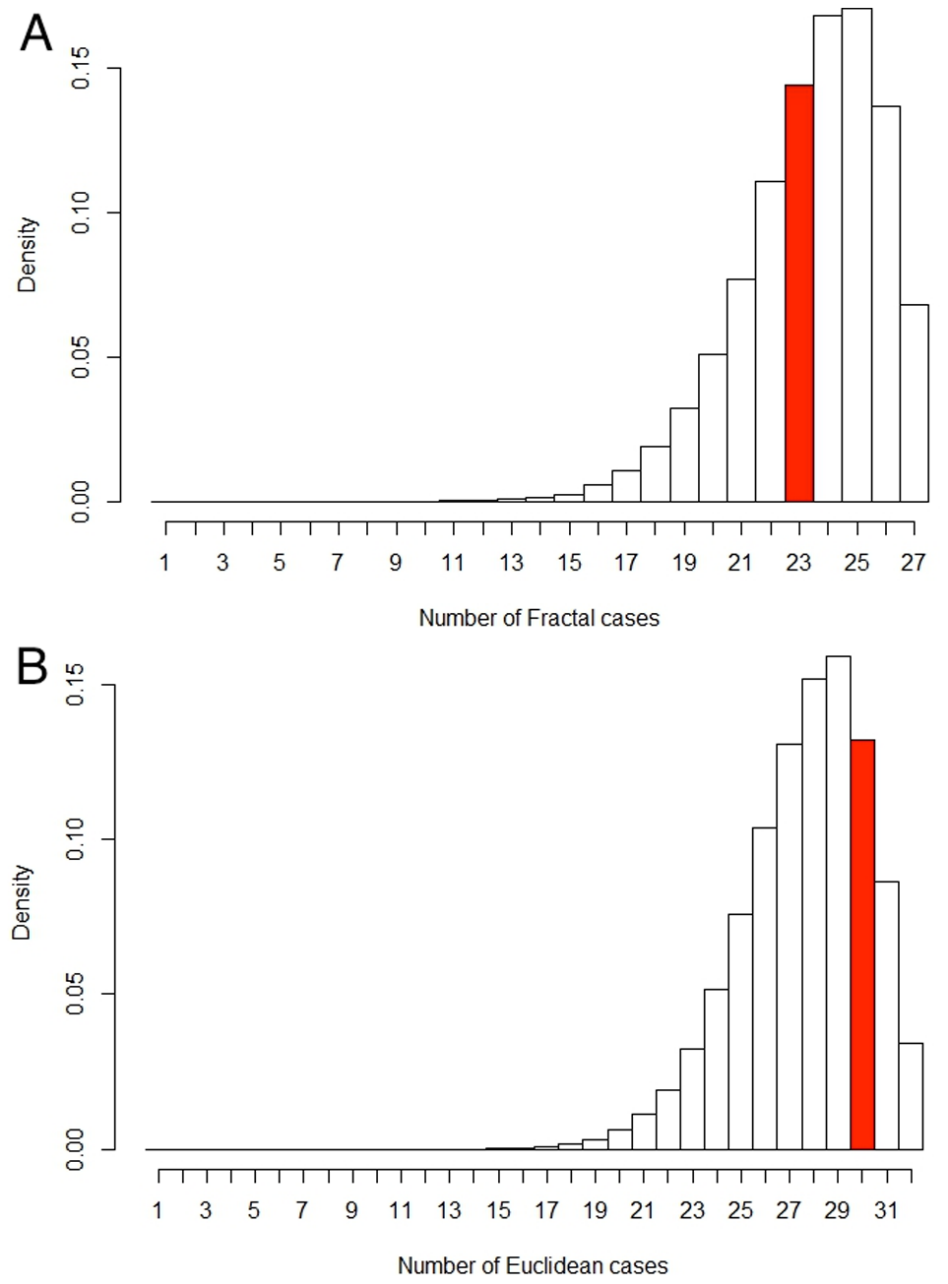
Density histograms of malignant/benign cases out of fractal/Euclidean cases. (A) Histogram of potential malignant cases out of 27 fractal cases given the posterior distribution *p*(*M*|*F*) (see Methods section). The red bar represents our testing data set of 23 malignant cases out of 27 total fractal cases. The probability of arriving at less than or equal to 23 cases is ∼46%. (B) Histogram of potential benign cases out of 32 Euclidean cases given the posterior distribution *p*(*B*|*E*). The red bar represents our testing data set of 30 benign cases out of a total of 32 Euclidean cases. The probability of arriving at less than or equal to 30 cases is ∼74%. Since 23 malignant cases (A) and 30 benign cases (B) are not towards either end of these distributions resulting from 100,000 “coin-toss” iterations, we can safely say that the respective models are an adequate fit for the data.

The Bayesian analysis presented above takes into account the limited size of the dataset and the robust statistical conclusions that are drawn from this analysis are indeed significant. Nevertheless, an additional statistical analysis was performed in order to further validate the significance of our results. Instead of constructing a statistical estimator and evaluating its frequency properties, a statistical model was developed by constructing a joint probability distribution and by checking it against the observational data, as suggested in [68]. A “coin toss” experiment was performed 100,000 times (see Methods section) to arrive at a discrete probability distribution, which showed that the model was an adequate representation of the data, as demonstrated by the red bars being at neither end of the distributions in Figures 7A and 7B. Indeed, the probability that the number of malignant cases (out of the 27 fractal cases) is less than 23, is ∼46%. Alternatively, the probability that the number of benign cases (out of the 32 Euclidean cases) is less than 30, is ∼74%. Both percentages (46% and 74%) being close to 50% is further validation that our results are not outliers in the model (e.g. <5% or >95%). Therefore, we can safely say that the model is an adequate fit for the data.

### Interpretation of the 3D Geometrical Structure

Even though two different 2D views of a 3D object are insufficient to fully characterize its 3D geometry, it can nonetheless give a robust estimate. The cases outside of the fractal zone can be categorized in two Euclidean subsets: 1) LINES, i.e. those that are approximately in the (*D*_CC_ = 1, *D*_MLO_ = 1) area, which are therefore seen a one-dimensional objects from both views (Fig. 6A); or 2) SHEETS, i.e. those that are either in the (*D*_CC_ = 1, *D*_MLO_ = 2) or (*D*_CC_ = 2, *D*_MLO_ = 1) areas, which are seen as a full two-dimensional object in one view, but as a one-dimensional object from the other view and also those that are in the (*D*_CC_ = 2, *D*_MLO_ = 2) area, which are seen as a full two-dimensional object from both views (Fig. 6B). Although simplistic, these case models represent a good estimate of what the 3D Euclidean structure of a benign lesion may look-like.

For the cases that fall in the fractal zone, those malignant lesions that are in the [1.2,1.8] × [1.2,1.8] square have a fractal signature that is seen from both views, whereas those that are in the triangular areas would represent fractal clusters that grow onto a 2D plane, i.e. seen as a fractal from one view, but seen either as a line (bottom or left triangular regions) or a plane (top or right triangular regions) from the other view (Fig. 6C). Interestingly, a diffusion-limited aggregate [69] embedded in 3D space and for which 2<*D*<3 will have a 2D projection with *D* = 2 [70]. Since no malignant lesions are found in the (*D*_CC_ = 2, *D*_MLO_ = 2) area of the CC-MLO fractal dimension plot, we can safely hypothesize that all tumors are essentially limited to a 2-dimensional fractal structure (within the 3-dimensional breast tissue), for which 1<*D*<2 [71], [72]. This therefore leads us to conjecture that all breast tumors considered in this study, benign and malignant, fractal or Euclidean, would grow on 2-dimensional manifolds.

## Conclusion & Discussion

Early detection and characterization of breast lesions are critical for the treatment and survival of the patient. Thus, the methodology presented here offers a way to accurately classify benign and malignant tumors based on their invasiveness determined by the geometrical structure obtained using this methodology. By considering the organization of tumors via CAD systems, current mammographic practice may be improved by increasing accuracy, and potentially decreasing recall rates and costs. The inferred 3-dimensional geometry of the breast lesions based on the analysis of the mammographic images using the 2D WTMM methodology allows us to explore the invasiveness of the breast tumors and provide an interpretation of the severity of the lesion. By considering where each case falls on the CC-MLO fractal dimension plot, a score similar to the BIRADs assessment score [14] may be assigned to each case. Not only does this tool have the potential as a CAD, but it may also provide insight into the underlying mechanisms that drive the overall growth and structure at the time of the screening mammogram. In this context, further analyses will help prove or disprove our conjecture that all MC clusters are restricted to growth on 2D manifolds within the 3D environment of the breast tissue.

Since the structure of the tumors are different, with benign lesions likely being smooth Euclidean objects and malignant lesions being branching objects (and possibly, for both cases, being restricted to growing along 2D manifolds within the 3D breast tissue environment), there may be a link to the cellular mechanisms at the lower levels in the system that drive the organization at the much larger scale of mammograms. There has been research done on the shape of cells and also the vascular structure feeding tumors [17], [73]. Studies such as these have shown the fractal structure of cancer at these levels, implying the global shape of the breast tumors may be driven by the interactions of smaller scale agents and their environment.

The nature of this study is exploratory. Future analyses on larger sets will allow the investigation of the automation of the selection process for the thresholds on *h* and on log(*K*). The delineation of the boundary between the fractal zone and the Euclidean zones in the CC-MLO fractal dimension plot, which was defined somewhat qualitatively, should also be explored. However, even though the size of the dataset investigated in this study is indeed limited, the robust statistical conclusions that are drawn from the analysis did take into account this limited sample size. Finally, the mathematical and computational approaches, as well as the results presented herein are novel and show great promise towards the establishment of a new paradigm in mammographic breast lesion detection and assessment.

## Methods

### Data

The images that were analyzed were obtained from the Digital Database for Screening Mammography (DDSM) at the University of South Florida [74], [75]. The databank contains over 2,600 studies made up of normal, benign, benign without call back and malignant mammograms all categorized by an expert radiologist. Each study has two images of each breast, consisting of a mediolateral-oblique (MLO) view and cranio-caudal (CC) view with any suspicious region circled by a radiologist. The suspicious region could contain a mass and/or microcalcifications (MC), but only the cases that were classified as having exactly one tumor composed of only microcalcifications in the benign and malignant categories were looked at in this particular study.

In addition to only considering tumors consisting of MC, any mammographic images that contained artifacts inside the radiologist's encircled region were discarded due to the impact it has on the analysis. These artifacts could include scratches, hair, deodorant, patient movement, scanner artifacts (rollers slipped), pacemaker, breast implants, skin markers (for scars, moles, and nipples, as well as marked lumps of breast pain), metallic foreign bodies, and fingerprints. Some (but not all) of these effects were recorded under notes in the DDSM website. More information on the extra care required to make efficient use of the DDSM as well as a general discussion on the next-generation open-access digital mammography library can be found in [76]. We thus considered a total of 59 cases corresponding to 118 images of size greater than 256^2^ pixels, 34 of which are benign (68 images) and 25 of which are malignant (50 images).

### 2D WTMM Method

Most of the existing CAD methods, whether specifically designed for (2D) mammograms [32], [33], [77]–[91] or more recently, for (3D) breast tomosynthesis [92]–[97], have been elaborated on the prerequisite that the background roughness fluctuations of normal breast texture are statistically homogeneous and uncorrelated, which precludes their ability to adequately characterize background tissue. The majority of the fractal methods used to examine and classify mammographic breast lesions rely on the estimate of the Hurst exponent (or its various mathematical equivalents), which globally characterizes the self-similar properties of the landscape in question. However, the 2D WTMM method takes in account that the function defining the image may be multifractal, therefore requiring the use of the Hölder exponent (Eq. (1)) to characterize the local regularity at a particular point [39], [40], [55], [56], [63].

The 2D WTMM method [39] requires us to define a smoothing function 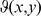 in two dimensions that is a well-localized isotropic function around the origin. In this application, we use the Gaussian function and define the wavelets as [64]

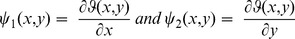
(2)


The wavelet transform with respect to ***ψ*** **_1_** and ***ψ***_2_ is
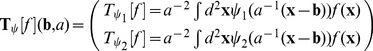
(3)

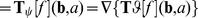
(4)from which we can extract the modulus and argument of the WT:
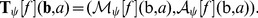
(5)where



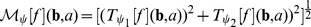
(6)




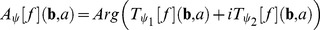
(7)


The wavelet transform modulus maxima are defined as the locations 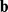 where 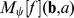 is locally maximum in the direction of the argument 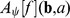 at a given scale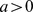. The WTMM lie on connected chains and are thus called maxima chains (Fig. 1) [39], [40], [55], [56]. Additional algorithmic details can be found in the Appendix of reference [43]. One can then find the maxima along these WTMM chains. The WTMM maxima, or WTMMM are defined as the points along the maxima chains where the 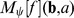 is locally maximum. The WTMMM are linked through scales to form the space-scale skeleton (Fig. 1G). 1G) point to in the limit *a* → 0^+^. Along these space-scale vertical lines the WTMMM behave as a power-law ∼*a^h^* ^(**x**)^ (Eq. (1)) from which one can extract the local Hölder exponent *h*(**x**). The multifractal formalism amounts to characterize the relative contributions of each Hölder exponent value via the estimate of the so-called *D*(*h*) singularity spectrum defined as the fractal dimension of the set of points **x** where *h*(**x**)  =  *h*. To compute *D*(*h*) we therefore use wavelets to partition the surface by defining the partition function directly from the WTMMM in the skeleton [39]:
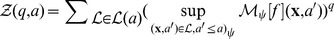
(8)where 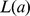 is the set of all vertical space-scale lines in the skeleton that exist at the given scale a>0 and which contain maxima at any scale 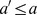 and 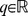. One can then define the exponent 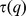 from the power-law behavior of the partition function:
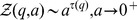
(9)and the 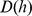 singularity spectrum of *f* can be determined from the Legendre transform of the partition function scaling exponent [39]

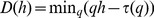
(10)


In practice, to avoid instabilities in the estimation of the singularity spectrum 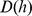 through the Legendre transform [98], [99], we use 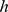 and 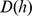 as mean quantities defined in a canonical ensemble, i.e. with respect to their Boltzmann weights computed from the WTMMM :
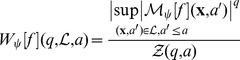
(11)


Then one computes the expectation values:
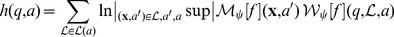
(12)and
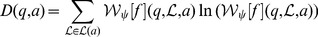
(13)from which one derives
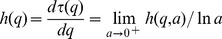
(14)

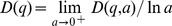
(15)and thus the singularity spectrum 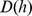 as a curve parameterized by *q*.

Homogeneous monofractal functions with singularities of unique Hölder exponent *H* are characterized by a linear 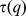 curve of slope *H*. A nonlinear 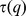 is the signature of nonhomogeneous multifractal functions, meaning that the Hölder exponent is a fluctuating quantity [39], [98], [99] that depends on **x**. Then the corresponding singularity spectrum has a characteristic single-humped shape. Note that for both mono- and multifractal functions
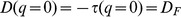
(16)where *D_F_* (noted simply *D* throughout the text) is the fractal dimension of the support of singularities of *f*.

### Statistical Tests

The Wilcoxon rank-sum test is a non-parametric statistical hypothesis test that is used as an alternative to Student's *t*-test when the population cannot be assumed to be normally distributed. It was used here to calculate the *p*-values comparing the CC and MLO fractal dimensions and benign and malignant cases images since the benign data followed a bimodal distribution (Fig. 4). The calculations were done using the Wilcox test in R.

### Bayesian Statistics

Bayes theorem states that
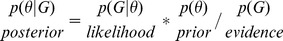
(17)where the prior, 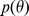, represents the strength of our belief in malignant lesions (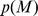) or benign lesions (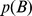) out of those that have been diagnosed by a radiologist. The posterior, 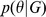, represents the strength of our belief, having accounted for the geometrical evidence, *G*, where *G* represents the position of the lesion in the fractal dimension plot, either fractal (*F*) or Euclidean (*E*). The quotient of the likelihood over the evidence, 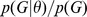, represents the support the evidence, *G*, provides for 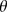. Since the prior reflects uncertainty in the parameter value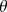, 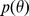
was based on a Beta distribution with a specified mean and standard deviation. The Beta distribution is as follows:
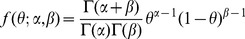
(18)


#### The Probability Model

To estimate the mean of the Beta distribution for malignant cases, 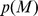, the prevalence of mammograms with a BI-RADS assessment score of 3, 4, and 5 were used as determined by the radiologists diagnostics multiplied by the historical probability of mammograms receiving those assessment scores result in malignant MC clusters respectively. One out of the 59 cases considered in this study received an assessment score of 3, 47 out of 59 received a 4, and 11 out of 59 received a 5. Based on historical data [14], [66], [67], the probability of malignant lesions given an assessment score of 3 is 2%, an assessment score of 4 is 26.5% (taken as the midpoint of the reported interval of [23%–30%], and an assessment score of 5 is 95%. Therefore the Beta distribution for 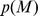 was chosen with a mean of
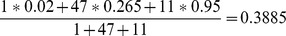
and the Beta distribution for 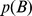 with a mean of 1–0.3885 = 0.6115. However, since there isn't much certainty regarding these values, the Beta distribution for 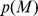 and 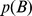 were assigned a relatively large standard deviation of 0.25. This resulted in a Beta distribution for 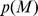 with parameters 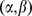  =  (1.09241,1.71223) and 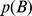 with parameters 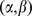  =  (1.71223,1.09241). The likelihood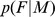, where *F* represents breast lesions characterized as being in the fractal zone (and *E* likewise represents those in the Euclidean zone), is based on the 23 of the 25 malignant cases that were in the fractal zone; this likelihood is the probability that the data could be generated with parameter values 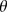. Similarly, the likelihood 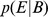 is based on the 30 out of 34 benign cases that were in the Euclidean zone. To arrive at the posterior distributions 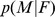 and 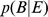, the R routine “BernBeta.R” was used, as defined in [65].

The resulting posterior distribution for 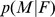 was a Beta distribution with parameters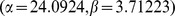. Based on this distribution, the resulting 95% highest density interval was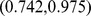. The posterior distribution for 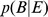 was a Beta distribution with parameters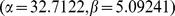. Based on this distribution, the resulting 95% highest density interval was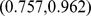. The highest density interval spans 95% of the posterior distribution such that every point inside the interval is deemed more credible. In other words, given the prior and the likelihood, observing the parameter value for the percentage of breast lesions characterized in the fractal zone that are malignant, there is a 95% probability that this parameter is between 0.742 and 0.975. Similarly, for the percentage of breast lesions characterized in the Euclidean zone that are benign, there is a 95% probability that this parameter is between 0.757 and 0.962.

### Statistical “coin toss” experiment

A random sample was taken from the posterior distribution, which gives a sample probability of a malignant case given fractal characterization, and similarly a benign case given Euclidean characterization. This probability was used to conduct the “coin toss” 27 times, which is the total number of cases in the fractal zone (23 malignant and 4 benign) and 32 times, which is the total number of cases in the Euclidean zones (30 benign and 2 malignant), all based on the posterior distribution. This random sampling was performed 100,000 times to arrive at a discrete probability distribution, i.e. a histogram, as shown in Fig. 7.
